# Anti-inflammatory mechanisms of IFN-γ studied in experimental autoimmune encephalomyelitis reveal neutrophils as a potential target in multiple sclerosis

**DOI:** 10.3389/fnins.2015.00287

**Published:** 2015-08-18

**Authors:** Nichole M. Miller, Jun Wang, Yanping Tan, Bonnie N. Dittel

**Affiliations:** BloodCenter of Wisconsin, Blood Research InstituteMilwaukee, WI, USA

**Keywords:** IFN-γ, multiple sclerosis, experimental autoimmune encephalomyelitis, neutrophils, anti-inflammatory

## Abstract

Multiple sclerosis (MS) is an autoimmune disease of the central nervous system (CNS) mediated by T helper (h)1 and/or Th17 CD4 T cells that drive inflammatory lesion development along with demyelination and neuronal damage. Defects in immune regulatory mechanisms are thought to play a role in the pathogenesis of MS. While an early clinical trial indicated that IFN-γ administration was detrimental to MS, studies in the mouse model of MS, experimental autoimmune encephalomyelitis (EAE), indicated that IFN-γ exhibits a number of anti-inflammatory properties within the CNS. These mechanisms include inhibition of IL-17 production, induction of regulatory T cells, T cell apoptosis and regulation of chemokine production. Mice deficient in IFN-γ or its receptor were instrumental in deciphering the anti-inflammatory properties of IFN-γ in the CNS. In particular, they revealed that IFN-γ is a major regulator of neutrophil recruitment into the CNS, which by a variety of mechanisms including disruption of the blood-brain-barrier (BBB) and production of reactive oxygen species are thought to contribute to the onset and progression of EAE. Neutrophils were also shown to be instrumental in EAE relapses. To date neutrophils have not been appreciated as a driver of MS, but more recently based largely on strong EAE data this view is being reevaluated by some investigators in the field.

## Introduction

Multiple sclerosis (MS) is an autoimmune disease driven by CD4 T cells of the T helper (h) 1 and/or Th17 subtype (Lovett-Racke et al., 2011). Once the pathogenic T cells enter the CNS they execute an inflammatory cascade that results in the infiltration of immune cells into the CNS from the periphery and the activation of local CNS antigen presenting cells, microglial cells, and astrocytes. This inflammatory response leads to loss of oligodendrocytes resulting in demyelination (Nylander and Hafler, 2012). It is now appreciated that neuronal damage/dysfunction starts at early stages of MS (Nylander and Hafler, 2012). Inflammation is a multifaceted process that requires cell-cell communication via cytokines and chemokines. The animal model of MS, experimental autoimmune encephalomyelitis (EAE), has greatly assisted with the process of deciphering the critical mediators of the inflammatory cascade that are important for CNS inflammation (McCarthy et al., 2012). The targeting of a number of cytokines and chemokines and their receptors has shown to alter EAE disease severity. One particular cytokine that has garnered much interest is interferon (IFN)-γ. While IFN-γ was first described in the context of viral infections, it quickly became clear that it exhibited pro-inflammatory properties and functioned more like an interleukin. While the root cause of MS is not clear, dysregulation of immune regulatory genes is thought to play a major role (Nylander and Hafler, 2012). In every inflammatory response it is essential that anti-inflammatory mechanisms must be turned on to avoid severe tissue damage (Dittel, 2008). Many immune regulatory effector molecules exhibit both pro- and anti-inflammatory functions (Dittel, 2008). IFN-γ is such an effector molecule and once genetic ablation was achieved in mice it was possible to unravel the anti-inflammatory mechanisms at play in the EAE model (Kelchtermans et al., 2008). Of particular interest to this review is that the pro-inflammatory properties of IFN-γ are dispensable for EAE onset. Rather it is the anti-inflammatory mechanisms that are the most critical by attenuating the severity of EAE. While many mechanisms have been proposed whereby IFN-γ exerts protection in EAE, in this review we concentrate on its role in regulating T cell subsets, apoptosis and suppressing neutrophil recruitment into the CNS. While neutrophils have not historically been considered a therapeutic target in MS, we believe that the prevailing evidence suggests that this perspective should be revisited.

## IFN-γ, a pleiotropic cytokine

In the early 1960s the existence of a soluble factor induced in human leukocytes with mitogens with anti-viral activity was known (Billiau and Matthys, 2009). In the early 1970s, it was clear that two distinct forms of interferons existed with the “classical interferons” termed Type I and the immune-induced interferon Type II (Billiau and Matthys, 2009). It wasn't until 1980 that the Type I interferons were named IFN-α and IFN-β and the Type II named IFN-γ (Billiau and Matthys, 2009). Today it is well established that IFN-γ's activities as a cytokine or interleukin are more important biologically than its anti-viral effects. The IFN-γ receptor (IFNGR) is a heterodimer composed of IFNGR1 and IFNGR2. IFN-γ ligand binding leads to receptor oligomerization and activation of the receptor associated Janus kinases (JAK) 1 and 2 that in turn phosphorylate the intracellular domain of IFNGR1 recruiting signal transducer and activator of transcription 1 (STAT1) (Ramana et al., 2002). The activation of STAT1 by phosphorylation results in its dimerization and translocation to the nucleus where it regulates gene transcription of IFN-γ-regulated genes (Ramana et al., 2002). While the primary producers of IFN-γ are T lymphocytes and natural killer (NK) cells, its receptor is expressed by virtually all cell types (Ramana et al., 2002; Platanias, 2005; Billiau and Matthys, 2009).

Biological activities of IFN-γ are broad and include: macrophage activation, upregulation of major histocompatibility complex (MHC) class I and II molecules, upregulation of antigen processing and presentation machinery, T helper (Th) 1 commitment of naïve CD4 T cells, inhibition of cell growth, apoptosis, regulation of leukocyte-endothelial interactions, and immunoglobulin isotype class switching, among other functions (Saha et al., 2010; Akdis et al., 2011). Thus, IFN-γ is a major contributor to both innate and adaptive immune responses. Mice deficient in either IFN-γ or the IFNGR1 subunit exhibit no developmental defects including normal hematopoiesis (Dalton et al., 1993; Huang et al., 1993). While mice lacking the IFNGR had normal cytotoxic and Th responses they exhibited increased susceptibility to *L. monocytogenes* and vaccinia virus (Huang et al., 1993). The same study also revealed the requirement for IFN-γ in IgG_2*a*_ production (Huang et al., 1993). IFN-γ-deficient mice were similar and were shown to have impaired macrophage activation, increased mitogen induced splenocyte proliferation, enhanced T cell cytolysis, and decreased NK cell activity (Dalton et al., 1993). Defective expression of the IFNGR in humans results in early susceptibility to mycobacterial infections (Zhang et al., 2008).

## Administration of IFN-γ exacerbated multiple sclerosis

Autoimmunity occurs when the immune system inappropriately targets self-tissue as foreign mounting an immune attack leading to extensive tissue damage that can lead to death. Autoimmunity can target any tissue or organ system and represents a large burden on the medical system with over 23 million Americans being affected. In addition, autoimmunity is one of the top 10 leading causes of death in women (Autoimmune Diseases Coordinating Committee, 2002). Multiple sclerosis (MS) is an autoimmune disease manifested in the central nervous system (CNS) that is considered both a demyelinating and neurodegenerative disease (Nylander and Hafler, 2012). The prevailing view is that CD4^+^ T cells are the initial drivers of inflammation in MS. In early studies using the animal model of MS, experimental autoimmune encephalomyelitis (EAE), IFN-γ producing Th1 cells were considered the pathogenic immune cell (Lovett-Racke et al., 2011).

Given that IFN-γ exhibits many immunoregulatory properties and since MS patients were reported to be deficient in its production a small clinical trial was performed to determine whether the administration of IFN-γ would be therapeutic (Neighbour et al., 1981; Vervliet et al., 1983; Panitch et al., 1987; Panitch, 1994). The results were unequivocal that IFN-γ therapy was not suitable for the treatment of MS given that it exacerbated disease. Fortunately, the patients completely recovered from the attacks (Panitch et al., 1987; Panitch, 1994). This result has set back future clinical trails targeting IFN-γ or its pathways even though, as discussed below, strong experimental data in EAE suggests that it functions as a potent anti-inflammatory mediator of CNS inflammation.

## Experimental evidence in EAE that IFN-γ is anti-inflammatory

Shortly after the MS IFN-γ clinical trail was reported EAE results examining the role of IFN-γ were observed in both the C57BL/6J and SJL/J models of EAE induced by immunization with spinal cord homogenate. Specifically, neutralizing antibody to IFN-γ increased both morbidity and mortality (Billiau et al., 1988). Conversely, systemic administration of IFN-γ reduced morbidity and mortality (Billiau et al., 1988). Similar findings were reported in the Lewis rat where systemic IFN-γ administration did not alter EAE induced by immunization with spinal cord homogenate, but suppressed disease when administered into the CNS ventricular system (Voorthuis et al., 1990). Furthermore, systemic administration of anti-IFN-γ just prior to disease onset exacerbated disease, while a single dose of IFN-γ given on day 8 of disease was sufficient to suppress disease (Voorthuis et al., 1990). In SJL/J mice the mild EAE that occurs following immunization with myelin basic protein (MBP) was greatly exacerbated following administration of two doses of anti-IFN-γ (Duong et al., 1992). When passive EAE was induced in SJL/J mice by the adoptive transfer of lymphocytes from MBP-challenged mice, anti-IFN-γ also resulted in severe EAE (Duong et al., 1992). Interestingly, while administration of anti-IFN-γ on day 0 drove more severe disease when given at later timepoints (day 7, 14, or 21) in the immunization model there was no impact on disease severity (Duong et al., 1992). Similar findings were observed in passive EAE (Duong et al., 1992). In a follow up study, this same group showed that anti-IFN-γ potentiated the development of EAE in a number of resistant mouse strains following immunization with MBP. In contrast to the human IFN-γ clinical trial, collectively the murine studies indicate that IFN-γ plays a protective role in EAE particularly early in disease likely due to effects in the periphery. In humans, IFN-γ was administered well after the onset of MS suggesting that in later stages of disease IFN-γ is a driver of disease perhaps via CNS specific mechanisms.

Once mice deficient in IFN-γ and its receptor were generated, the above EAE studies were repeated with similar findings. For the IFN-γ-deficient experiments the knockout was backcrossed onto the B10.PL susceptible strain. When EAE was induced by immunization with guinea pig MBP disease onset was similar in +∕+ and −∕− littermate controls, but while the WT underwent recovery the knockout mice exhibited a high mortality rate (Ferber et al., 1996) (Table 1). For the IFNGR-deficient study the mice used were the EAE resistant 129/Sv mouse strain. Upon immunization with the human myelin oligodendrocyte glycoprotein (MOG)_35−55_ peptide, EAE occurred in 100% of the deficient animals (Willenborg et al., 1996) (Table 1). In passive EAE, while both the +∕+ and −∕− littermates were equally susceptible to EAE, only the knockout mice exhibited mortality (Willenborg et al., 1999) (Table 1). A similar finding indicating that IFN-γ conferred resistance to EAE was also reported in IFN-γ^−∕−^ BALB/c mice (Krakowski and Owens, 1996) (Table 1). The above studies clearly demonstrated that IFN-γ plays a protective role in EAE initiating a multitude of subsequent studies focused on specific mechanisms.

**Table 1:** **Summary of EAE disease susceptibility and presentation in various mouse strains deficient in IFN-γ or its receptor**.

**Mouse strain^a^**	**EAE mouse^b^**	**EAE induction^c^**	**EAE disease**	**References**
B10.PL; susceptible	IFN-γ^–/–^	Active: guinea pig MBP	Severe with high mortality	Ferber et al., 1996
129/Sv; resistant	IFNGR^–/–^	Active: human MOG_35−55_	High susceptibility, severe disease	Willenborg et al., 1996
129/Sv; resistant	IFNGR^–/–^	Passive: human MOG_35−55_	High mortality	Willenborg et al., 1999
BALB/c; resistant	IFN-γ^–/–^	Active: bovine MBP	High susceptibility, severe disease	Krakowski and Owens, 1996
BALB/c; resistant	IFN-γ^–/–^	Passive: MBP exon 2	High susceptibility, high atypical disease	Abromson-Leeman et al., 2004
MBP-TCR B10.PL, susceptible	IFN-γ^–/–^Rag2^–/–^	Spontaneous, mouse MBP_Ac1−11_	Atypical	Wensky et al., 2005
B10.PL; susceptible	IFN-γ^–/–^	Passive: mouse MBP_Ac1−11_	Classical	Wensky et al., 2005

a *Indicates the mouse strain in which EAE was induced and whether the WT mouse is susceptible to EAE*.

b *Indicates the gene deficiency status of the mouse in which EAE was induced*.

c *Indicates whether EAE was induced by active immunization with the antigen used indicated or by passive induction by the adoptive transfer of encephalitogenic T cells with their specificity indicated*.

## Influence of IFN-γ on the effector CD4 Th phenotype

Naïve CD4 T cells are plastic and with the provision of specific cytokine stimuli differentiate into unique Th subsets. The quintessential pathways are Th1, Th2, and more recently Th17. Th1 cells require IL-12 signaling and express IFN-γ in the absence of IL-4 production. While IL-12 is not required for the induction of EAE, its administration at disease initiation, but not onset, was shown to suppress EAE via the induction of IFN-γ (Zhang et al., 2003; Gran et al., 2004). Th2 cells express IL-4 and also require it for differentiation. Th2 cells are not encephalitogenic and interestingly their differentiation is blocked by IFN-γ. We have shown that IL-4 production within the CNS is protective during EAE. However, whether IFN-γ downregulates CNS derived IL-4 is not known (Ponomarev et al., 2007a). More recently, CD4 T cells that produce IL-17 have been described with encephalitogenic potential (Lovett-Racke et al., 2011). IL-17 production by CD4 T cells requires TGF-β and IL-6 signals and expression of the transcription factor RORγt. IFN-γ has been shown to inhibit IL-17 production by CD4 T cells and in its absence Th17 cells are increased in the CNS during EAE (Harrington et al., 2005; Park et al., 2005; Berghmans et al., 2011). However, IL-17 is not an essential cytokine for the induction or progression of EAE (Komiyama et al., 2006). Along with our colleagues, we showed that the critical cytokine produced by both Th1 and Th17 cells for the induction of EAE is GM-CSF (Ponomarev et al., 2007b; El-Behi et al., 2011). As with IFN-γ, GM-CSF is also a pleiotropic cytokine that can influence EAE induction by a variety of mechanisms including activation of antigen presenting cells, enhancement of pro-inflammatory cytokine production and promotion of leukocyte adhesion and chemotaxis (Shiomi and Usui, 2015). In addition, GM-CSF can circumvent negative regulation by IFN-γ by inhibiting STAT1 tyrosine phosphorylation with the net result being reduced IFN-γ-induced gene transcription (Kasper et al., 2007). Thus, by controlling the phenotype of naïve T cells with encephalitogenic potential IFN-γ influences the initiation and severity of EAE.

## Influence of IFN-γ on the regulatory CD4 phenotype

It is well established that an important tolerance mechanism keeping autoimmune responses in check are CD4 T cells that express the transcription factor Foxp3. Mice and humans with mutations in Foxp3 quickly succumb to a lethal autoimmune syndrome (Bennett et al., 2001; Brunkow et al., 2001; Wildin et al., 2001; Fontenot et al., 2003; Hori et al., 2003). CD4^+^Foxp3^+^ T regulatory cells (Treg) that develop in the thymus are termed natural (n) Treg (Fontenot et al., 2003; Hori et al., 2003). Treg can also be induced (i) in the periphery in the presence of TGF-β and IL-2 (Chen et al., 2003; Davidson et al., 2007). In EAE, Treg have been shown to strongly inhibit both the onset and progression of disease (Kohm et al., 2002; McGeachy et al., 2005; Gultner et al., 2010; Ray et al., 2012). IFN-γ does not seem to influence nTreg development given that IFNGR^−∕−^ mice were shown to harbor normal numbers of functional Treg (Kelchtermans et al., 2005). Rather IFN-γ promotes iTreg conversion by inducing Foxp3 expression generating iTreg with the capacity to suppress the severity of EAE (Wang et al., 2006).

IL-27 also induces a Treg phenotype that were shown to be distinct from the iTreg induced by IFN-γ (Hall et al., 2012). These cells termed Tr1 express IFN-γ and high levels of IL-10 and play an important role in controlling local inflammation induced by Th1 cells, particularly during Toxoplasmosis and Leishmaniasis (Anderson et al., 2007; Jankovic et al., 2007; Hall et al., 2012). IL-10 is a potent anti-inflammatory cytokine that has been shown to be suppressive in EAE (Bettelli et al., 1998). Tr1 cells generated *in vitro* with IL-27 and IL-12 stimulation from MOG_35−55_ immunized mice exhibited less encephalitogenic potential as compared to CD4^+^ T cells stimulated in IL-12 alone (Apetoh et al., 2010). In addition to generating Tr1 cells, CD27 can also potentially limit EAE severity by blocking Th17 development (Fitzgerald et al., 2007; Diveu et al., 2009; Sweeney et al., 2011). IL-27 was shown to block Th17 development by suppressing RORγt expression in a STAT1-dependent manner (Diveu et al., 2009). Interestingly, MS patients that do not respond to IFN-β therapy were shown to produce lower levels of IL-27 following stimulation with IFN-β than the responder patients (Sweeney et al., 2011). Thus, IL-27 production is an important negative regulator of CNS inflammation by inducting Tr1 cells that express the anti-inflammatory cytokines IL-10 and IFN-γ, while limiting Th17 cell generation.

## Role for IFN-γ in controlling T cell apoptosis

In 1990 IFN-γ was implicated in T cell death in a study showing that anti-IFN-γ blocked the death of a cloned Th1 cell line stimulated via the TCR in the absence of co-stimulation (Liu and Janeway, 1990). From a number of studies, it is clear that limiting the number of encephalitogenic CD4 T cells within the CNS can either prevent or greatly reduce the severity of EAE. This has been clearly demonstrated in IFN-γ^−∕−^ mice, which were shown to have a significant increase in the number of activated CD4 T cells 21 days following EAE induction (Chu et al., 2000). These T cells were undergoing increased proliferation and decreased apoptosis, demonstrating an *in vivo* a role for IFN-γ in the induction of CD4 T cell apoptosis (Chu et al., 2000). Subsequent studies demonstrated that the proapoptic effects of IFN-γ could be mediated by the induction of caspase-1 by the interferon regulatory factor (IRF)-1 transcription factor in a STAT1-dependent manner (Chin et al., 1997). Conversely, caspase-1 plays a role in EAE pathogenesis by cleaving pro-IL-1β as a component of the nucleotide-binding oligomerization-like receptor family, pyrin domain containing 3 (NLRP3) inflammasome. IL-1β can regulate EAE by several mechanisms. First, it can upregulate VCAM-1 expression by endothelial cells, which is a ligand for VLA-4 an essential integrin required for CD4 T cell migration into the CNS (Baron et al., 1993). It was recently shown that CD4^+^ T cells require priming by NLRP3 inflammasome-sufficient antigen presenting cells to obtain the ability to migrate into the CNS via upregulation of specific chemokine receptors (Inoue et al., 2012). A second important mechanism for IL-1β is its promotion of IL-17 production by CD4 and γδ T cells in synergy with IL-6 and IL-23 (Chung et al., 2009; Lalor et al., 2011). In addition to its effects on CD4 T cells, caspase-1 also plays a detrimental role in oligodendrocyte injury and demyelination (Ren et al., 2011; Loda and Balabanov, 2012). Thus, IFN-γ via its upregulation of caspase-1 plays both protective and pathogenic roles in CNS inflammation.

In addition, IFN-γ has the capacity to influence Fas-dependent cell death by increasing the surface expression of Fas, also in a STAT1-dependent manner (Xu et al., 1998). Interestingly, in the later study, IFN-γ-induced cell death was inhibited by caspase 1 inhibitors (Xu et al., 1998). We, and others, have clearly demonstrated a role for Fas expression within the CNS for the onset of EAE (Dittel et al., 1999; Sabelko-Downes et al., 1999; Dittel, 2000). In addition, FasL expression by encephalitogenic T cells was shown to play a pathogenic role in EAE induction (Dittel et al., 1999; Sabelko-Downes et al., 1999; Dittel, 2000). However, in our study, we did not find a role for Fas expression by the encephalitogenic T cells in regulating the severity of EAE (Dittel et al., 1999). A subsequent study using conditional knockout mice with an oligodendrocyte deficiency in Fas, demonstrated its role in inducing demyelination (Hovelmeyer et al., 2005).

An additional mechanism whereby IFN-γ can induce T cell death is via the induction of indoleamine 2,3 dioxygenase (IDO) in dendritic cells (Mellor and Munn, 2004). Through its degradation of tryptophan, an essential amino acid required for T cell proliferation, IDO promotes T cell death (Mellor and Munn, 2004). A number of studies have demonstrated IDO expression in the CNS in astrocytes and microglia/macrophages and its importance in the suppression of EAE (Sakurai et al., 2002; Kwidzinski et al., 2003, 2005; Yan et al., 2010). Tryptophan metabolites generated by IDO have been shown to promote Treg generation, which is an additional mechanism whereby IFN-γ controls CD4 T cell phenotypes (Yan et al., 2010).

## The role of IFN-γ inducible chemokines in EAE

In 1998 Drs. Karpus and Ransohoff wrote an insightful commentary suggesting that temporal and spatial chemokine expression in the CNS during EAE governed disease pathogenesis (Karpus and Ransohoff, 1998). Evidence supporting this hypothesis will be discussed. Largely by the use of chemokine receptor knockout mice, the role for specific chemokines in the more severe disease observed in mice deficient in IFN-γ signaling has been revealed. As discussed above, IFN-γ can promote the migration of T cells into the CNS by upregulating chemokine receptors and adhesion molecules. In addition, IFN-γ can influence cell migration into the CNS by either upregulating or inhibiting specific chemokine production.

Two IFN-γ-inducible chemokines that were hypothesized to be involved in EAE initiation and severity are CXCL9 (MIG) and CXCL10 (IP-10) (Karpus and Ransohoff, 1998). Both chemokines along with CXCL11 (I-TAC), which is also IFN-γ-inducible, share CXCR3 as their receptor, which is preferentially expressed by Th1 cells (Sallusto et al., 1998). Differential expression of CXCR3 by Th17 cells has been reported and may depend on whether they also produce IFN-γ (Jadidi-Niaragh and Mirshafiey, 2011). CXCL9, CXCL10, and CXCL11 have been shown to be upregulated in the CNS during EAE, in a manner consistent with IFN-γ production by Th1 encephalitogenic T cells (Carter et al., 2007; Kroenke et al., 2008). In EAE, while CXCR3^−∕−^ mice exhibited more severe EAE there was no alteration in the gross quantitative and qualitative nature of the inflammatory infiltrates (Liu et al., 2006; Muller et al., 2007). However, CD4^+^Foxp3^+^ Treg were shown to be reduced in the white matter of CXCR3^−∕−^ mice (Muller et al., 2007). CNS tissues from CXCR3^−∕−^ mice exhibited more severe blood-brain-barrier (BBB) disruption and reduced levels of IFN-γ in the serum and spinal cord (Liu et al., 2006). A role for CXCL9 has not been carefully examined in EAE. CXCL10 has been shown to be pathogenic in EAE in several studies that neutralized CXCL10 with blocking antibodies (Fife et al., 2001; Narumi et al., 2002). In contrast, in later studies neither anti-CXCL10 nor a global deficiency in CXCL10 resulted in more severe EAE (Klein et al., 2004; Tsunoda et al., 2004; Byrne et al., 2009). However, CXCL10^−∕−^ mice were more susceptible to EAE onset when induced with subthreshold levels of MOG_35−55_ (Klein et al., 2004). The above cumulative studies suggest that CXCL9 and CXCL10 are not critical cytokines in the induction of EAE and in the most recent study examining this issue EAE severity induced by MOG_35−55_ immunization was not altered in CXCR3^−∕−^ or CXCL10^−∕−^ mice (Lalor and Segal, 2013). C57BL/6 mice do not produce functional CXCL11 so its role in EAE was investigated using CXCL11-Ig that upon adoptive transfer suppressed EAE (Zohar et al., 2014). CXCL11 is thought to be protective in EAE via the induction of Tr1 regulatory T cells (Zohar et al., 2014). CXCL10-Ig had no effect (Zohar et al., 2014). Further evidence for a protective role for CXCL11 was demonstrated using as CXCR3 receptor antagonist composed of a truncated CXCL11(4-79) (Kohler et al., 2008). Thus, there is little evidence that IFN-γ-inducible chemokines play a major pathogenic role in EAE. This is likely due to redundancy in the chemokine family whereby encephalitogenic T cells can utilize chemokine receptors other than CXCR3 to migrate into the CNS.

## IFN-γ regulates the EAE clinical phenotype

One of the first reports to examine chemokine production in mice deficient in IFN-γ reported that CCL2 (MCP-1) and CXCL1 (Gro-α), but not CXCL10 were expressed in the CNS (Glabinski et al., 1999). The next year, Tran et al., addressed the same question and found that CCL5 (RANTES) and CCL2 were not detectable in the CNS of IFN-γ^−∕−^ or IFNGR^−∕−^ mice with EAE (Tran et al., 2000). In contrast, CXCL2 (MIP2β, GRO-β) and CCL1 (TCA-3) were upregulated in the same mice. Both of the later chemokines attract neutrophils, which is consistent with a predominance of Gr-1^+^ neutrophils in the CNS of both IFN-γ^−∕−^ and IFNGR^−∕−^ mice with EAE (Tran et al., 2000). It is now known that Gr-1 antibodies recognize Ly6C expressed by monocytes/macrophages and neutrophils and Ly6G expressed by neutrophils. Thus, Gr-1 recognition over estimated the number of neutrophils in the lesions. However, H&E staining did indicate an increased presence of cells with a PMN morphology in both IFN-γ^−∕−^ and IFNGR^−∕−^ mice with EAE (Willenborg et al., 1996; Tran et al., 2000).

It is now well established that a deficiency in IFN-γ or its receptor has a profound effect on the nature of the EAE disease course. Classical EAE in WT presents as an ascending paralysis with inflammatory lesions in the white matter of the spinal cord (Cross et al., 1993). Non-classical or atypical disease presents with distinct clinical signs consisting of a slight head tilt that progresses to a severe head tilt or with the mouse laying on its side, which subsequently leads to spinning or an axial rotatory motion (Abromson-Leeman et al., 2004; Wensky et al., 2005). This atypical disease was associated with predominate lesions in the brain, cerebellum and brainstem (Abromson-Leeman et al., 2004; Wensky et al., 2005) (Table 1). The first report of atypical disease was observed in Balb/c IFN-γ^−∕−^ mice immunized with MBP peptides (Abromson-Leeman et al., 2004). Lesions in both the classical and atypical EAE contained neutrophils as determined by histology (Abromson-Leeman et al., 2004) (Table 1). EAE induced by adoptive transfer indicated a role for the encephalitogenic T cell in determining whether disease presented as classical or atypical (Abromson-Leeman et al., 2004) (Table 1). The second report describing atypical disease utilized transgenic B10.PL mice bearing a TCR specific for myelin basic protein (MBP) (MBP-TCR), which succumb to spontaneous EAE with a 100% penetrance when crossed to Rag1^−∕−^ mice (Goverman et al., 1993; Wensky et al., 2005). As in earlier reports, mice lacking IFN-γ^−∕−^ had a prominence of granulocytes in the CNS lesions (Wensky et al., 2005). In the same study, EAE induced by the adoptive transfer of Th1 MBP-TCR T cells into IFN-γ^−∕−^ mice resulted in classical disease, supporting the notion that encephalitogenic T cells play an essential role in determining the nature of EAE disease (Wensky et al., 2005). This was further explored in two studies published in 2008, which specifically compared a role for Th1 and Th17 encephalitogenic T cells using adoptive transfer. In the first study, Th17 skewed encephalitogenic T cells induced predominantly atypical EAE with Th1 driving classical disease (Stromnes et al., 2008). Furthermore, it was concluded that it was not the number of Th17 cells that drove brain lesions, but their predominance over Th1 cells that was important (Stromnes et al., 2008). A specific role for IL-17 production was indicated by the loss of inflammatory lesions in the brain parenchyma, but not spinal cord, upon injection of anti-IL-17 (Stromnes et al., 2008). The ratio of Th1:Th17 was shown to be important in determining lesion localization with the conclusion that inflammation in the brain parenchyma occurred when Th17 were more prevalent leading to a disproportionate increase in IL-17 in the brain (Stromnes et al., 2008). Of interest, anti-IL-17 also reduced neutrophil infiltration in the spinal cord. In a second study, it was also found that increasing numbers of Th17 T cells was not sufficient to drive atypical EAE (Lees et al., 2008). In addition, IFN-γ^−∕−^ encephalitogenic T cells were shown to predominantly induce classical disease indicating that IFN-γ production by T cells is not the driving factor in regional localization (Lees et al., 2008). While granulocyte infiltration was not specifically examined, an increase in CXCL2 was observed in the spinal cord of mice with severe Th1 disease as compared to the cerebellum (Lees et al., 2008).

## IFN-γ regulation of EAE occurs in both the CNS and peripheral immune cells

**Figure 1:**
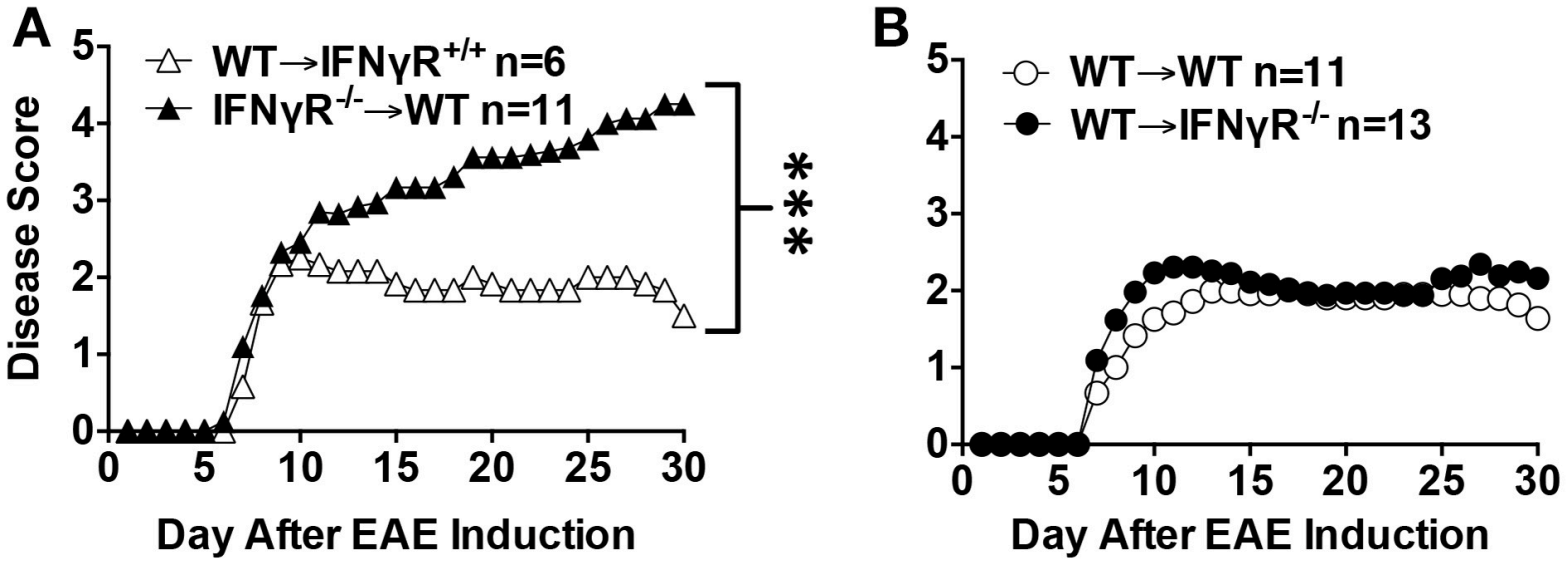
**A deficiency in the IFNGR in the peripheral immune system, but not the CNS, leads to severe EAE**. B10.PL IFNGR^−∕−^ mice were generated by crossing C57BL/6 IFNGR^−∕−^ mice to B10.PL for two generations and then intercrossing. BM chimera mice were generated by transplanting either WT **(A,B)**, IFNGR^+∕+^ **(A)** or IFNGR^−∕−^ **(B)** littermates with donor BM from either WT **(A,B)** or IFNGR^−∕−^ **(A)** mice as described (Ponomarev et al., 2005). EAE was induced by the adoptive transfer of WT 1 × 10^6^ MBP-TCR encephalitogenic T cells as described (Dittel et al., 1999). EAE was scored daily on a five point scale: 0-no disease, 1-tail weakness, 1.5-hind limb ataxia, 2-hind limb paresis, 2.5-one hind limb paralyzed, 3-both hind limbs paralyzed, 4-hind and forelimbs paralyzed, 5-death. The data are the mean daily score from two experiments. ^***^p < 0.001 by the Mann-Whitney test.

A limiting factor of the use of global knockouts is that they did not allow the discrimination of whether the IFN-γ target were cells within the CNS or periphery. This question was addressed by the generation of bone marrow (BM) chimeras and cell specific deficiencies. The chimera strategy was utilized first by transplanting IFNGR^−∕−^ BM into WT mice (IFNGR^−∕−^ → WT) generating mice in which only the peripheral immune system lacks IFN-γ signaling. In the reverse chimera (WT → IFNGR^−∕−^) because CNS resident cells including microglial cells do not turn over after transplantation the CNS along with non-hematopoeitic peripheral cells are deficient in the IFNGR (Ponomarev et al., 2005). In the first study to utilize this strategy, encephalitogenic T cells were derived from MOG_35−55_ immunized IFNGR^−∕−^ mice (Willenborg et al., 1999). The authors concluded that IFNGR regulation occurred in both the hematopoietic lineage and in the host tissues with no discussion of atypical disease (Willenborg et al., 1999). A second study utilizing an identical approach except that WT encephalitogenic T cells were used came to the same conclusion with the addition that both chimeras exhibited atypical disease, which was more prevalent in the IFNGR^−∕−^ → WT chimeras (Lees et al., 2008). The opposite result was observed in a third study whereby atypical disease was more prevalent in WT → IFNGR^−∕−^ chimeras (Lee et al., 2012). The reason for the difference in results is not clear, but since EAE was induced by immunization with MOG_35−55_ in the later study the encephalitogenic T cells were not equivalent in regards to IFN-γ responsiveness, which could influence whether they are more Th1- or Th17-like (Lee et al., 2012). We addressed the issue of the IFN-γ-responsive cell in our own studies whereby EAE was induced in BM chimeras by adoptive transfer of WT MBP-TCR encephalitogenic T cells (Dittel et al., 1999; Ponomarev et al., 2005). We found that IFNGR^−∕−^ → WT chimeras exhibited progressive disease (Figure 1A), while no difference in the EAE disease course was observed in the WT → IFNGR^−∕−^ chimeras (Figure 1B) as compared to control WT chimeras. Atypical disease was observed only rarely in our studies (data not shown). From our studies, we conclude that the IFN-γ-responsive cell regulating typical EAE resides within the peripheral immune system.

One drawback to the WT → IFNGR^−∕−^ chimeras is that a deficiency in IFN-γ responsiveness is not restricted to the CNS, thus it is possible that IFN-γ regulates EAE severity and regionalization from the periphery. To circumvent that issue transgenic mice were generated with a signaling defective dominant-negative IFNGR specifically in astrocytes (Hindinger et al., 2005, 2012). When EAE was induced by immunization with MOG_35−55_, the transgenic mice exhibited a chronic slightly progressive disease as compared to WT mice with no mention of atypical disease (Hindinger et al., 2012). Further analysis implicated a role for IFN-γ in dampening astrocyte activation that could limit their production of chemokines and pro-inflammatory cytokines (Hindinger et al., 2012). A second approach to the same question silenced IFNGR signaling in either astrocytes or microglial cells using lentiviral vectors that expressed IFNGR1-specific shRNA using GFAP and CD11b to drive their expression, respectively. The lentiviral vectors were i.c.v. injected 3 days prior to EAE induction by immunization with MOG_35−55_ (Ding et al., 2015). When the IFNGR was silenced in astrocytes, EAE severity was significantly decreased and was accompanied by decreased immune cell infiltrates (Ding et al., 2015). A similar result was obtained in both Th1 and Th17 adoptive transfer EAE (Ding et al., 2015). The administration of the GFAP silencing vector after disease onset also attenuated disease severity (Ding et al., 2015). It was speculated that the difference in outcome between the two astrocyte-focused studies was due to the dominant-negative receptor serving as an IFN-γ sink limiting its availability to negatively regulate EAE via other cell types (Ding et al., 2015). When CD11b^+^ cells were silenced, EAE was more severe with no mention of atypical EAE (Ding et al., 2015). While the authors contributed this to silencing in microglial cells, they did not definitively rule out a role for peripheral immune CD11b^+^ cells in the negative regulation (Ding et al., 2015). Cumulatively, the above studies indicate that both CNS resident and peripheral immune cells play a role in IFN-γ-mediated regulation of EAE and that atypical EAE is variable depending upon the EAE disease model.

## Neutrophils are essential drives of EAE pathogenesis

In the articles discussed above one recurrent theme in mice deficient in IFN-γ or its receptor is the presence of granulocytes and an increase in chemokines that recruit them termed ELR^+^ CXC chemokines. An essential role for granulocytes was demonstrated by a dramatic reduction in EAE incidence induced by either active immunization or adoptive transfer in mice treated with anti-Gr-1 (RB6-8C5) or with anti-Ly6G (1A8) (McColl et al., 1998; Carlson et al., 2008; Steinbach et al., 2013). CXCL1 and CXCL2 are prototypic ELR^+^ CXC chemokines that bind CXCR2 expressed by granulocytes. EAE induced in SJL/J mice with proteolipid protein (PLP)_139−151_ led to a significant increase in CXCL1 and CXCL2 in the spinal cord as early as day 8–9 following immunization, which coincided with the appearance of CXCR2^+^ cells (Carlson et al., 2008). An essential role for CXCR2^+^ cells in EAE induction was demonstrated by reduced EAE following antibody blocking or complete protection from disease with genetic ablation of CXCR2 (Carlson et al., 2008). The transfer of CXCR2^+^ cells into CXCR2^−∕−^ mice was sufficient to restore EAE susceptibility (Carlson et al., 2008). It was concluded that granulocytes drove disease by disruption of the blood-brain-barrier (BBB) without altering peripheral T cell responses (McColl et al., 1998; Carlson et al., 2008; Christy et al., 2013; Steinbach et al., 2013). An alternative mechanism whereby granulocytes attenuate EAE was speculated to be the induction of iNOS in macrophages/neutrophils (Gr-1^hi^) by T cell-derived IFN-γ that in turn suppressed T cell proliferation. However, this has not been confirmed *in vivo* within the CNS (Willenborg et al., 1999; Zehntner et al., 2005). Neutrophils have also been implicated in the maturation of APC within the CNS via their production of pro-inflammatory cytokines (Steinbach et al., 2013).

Interestingly, granulocyte depletion also inhibited EAE onset in IFNGR^−∕−^ mice (McColl et al., 1998). In back-to-back papers the Segal and Goverman laboratories tied together regional production of ERL^+^ CXC chemokines and the penetrance of atypical EAE (Simmons et al., 2014; Stoolman et al., 2014). In the Segal paper, atypical EAE in C57BL/6 IFNGR^−∕−^ mice was associated with the upregulation of CXCL2 in the brainstem and the recruitment of neutrophils into the site in a CXCR2-dependent manner (Stoolman et al., 2014). In classical EAE in WT mice, CCL2 was increased in the spinal cord driving the recruitment of monocytes/macrophages in a partially CCR2-dependent manner (Stoolman et al., 2014). Given that IL-17 induces CXCL2 the conclusion that atypical EAE was IL-17-independent is an important finding (Iwakura et al., 2011; Stoolman et al., 2014). In the Goverman study using C3HeB/FeJ mice deficient in either IFNGR or IL-17RA, it was shown that IL-17 promoted ELR^+^ CXC chemokine-mediated recruitment of neutrophils into the brain while IFN-γ inhibited ELR^+^ CXC chemokine and neutrophil production in the brain, but had the converse effect in the spinal cord (Simmons et al., 2014). In C3HeB/FeJ mice, IL-17 was shown to play a more prominent role in driving atypical disease (Simmons et al., 2014).

**Figure 2:**
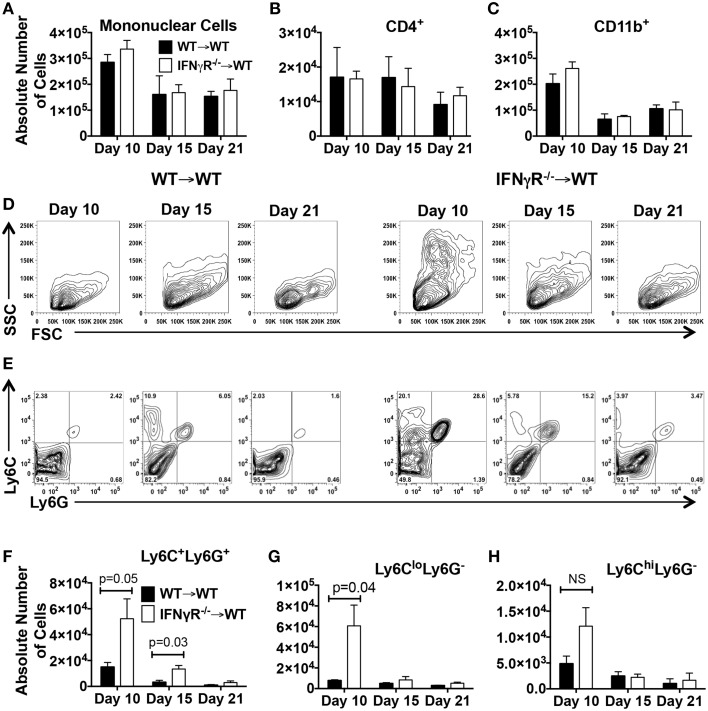
**Mice deficient in the IFNGR in the peripheral immune system do not have increased numbers of mononuclear, CD11b^+^ or CD4^+^ cells in the CNS during EAE, but have significant increases in neutrophils and macrophages**. BM chimera mice were generated by transplanting WT mice with donor BM from either WT (WT → WT) or IFNGR^−∕−^ (IFNGR^−∕−^ → WT) mice as described (Ponomarev et al., 2005). On days 10, 15, and 21 after EAE induction total mononuclear cells were isolated and counted on a hemacytometer and subsequently analyzed by flow cytometry for CD45 (total mononuclear cells) **(A)**, CD4 (CD45^+^CD11b^−^CD4^+^) **(B)**, CD11b (CD45^+^CD11b^+^) **(C)**, neutrophils (CD11b^+^Ly6C^+^Ly6G^+^) **(F)**, microglial cells and resident macrophages (CD11b^+^Ly6C^lo^Ly6G^−^) **(G)** or inflammatory macrophages (CD11b^+^Ly6C^hi^Ly6G^−^) **(H)** (Ponomarev et al., 2005). Each time point is the mean ± SD of three experiments with pooled cells from 2 to 4 mice. Representative FSC and SSC light scatter profiles **(D)** and counter plots of CD11b-gated cells for expression of Ly6C and Ly6G **(E)** on days 10, 15, and 21 are shown for WT → WT and IFNGR^−∕−^ → WT chimeras.

**Figure 3:**
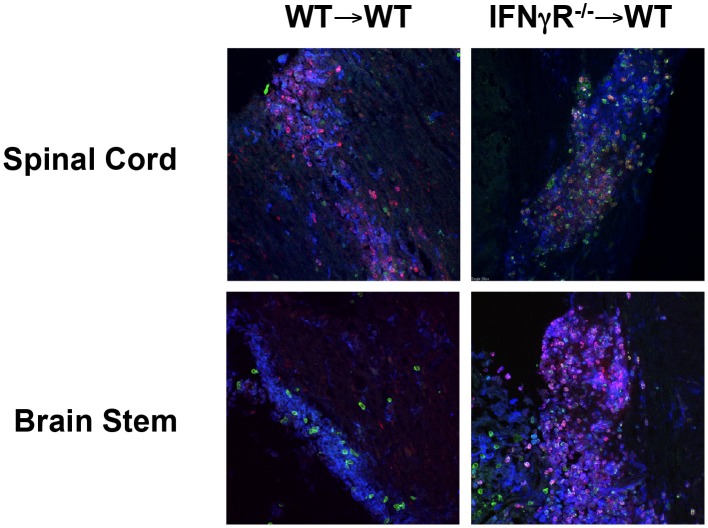
**Neutrophils are present in spinal cord lesions in both WT → WT and IFNGR^−∕−^ → WT chimeras, but are only present in the brain stem of IFNGR^−∕−^ → WT chimeras**. The generation of BM chimeras and EAE induced were performed as for Figures 1, 2, respectively. On day 10 after EAE induction mice were perfused and frozen sections (10 μm) of the spinal cord and brain stem were generated as described (Shriver and Dittel, 2006). The sections were stained with anti-mouse CD11b (blue), anti-mouse TCRβ (green), and anti-mouse Ly6C (red) and visualized by confocal microscopy. Scale bar: 20 μm.

The above Segal and Goverman studies utilized global knockout mice thus it was not possible to discern the location of the IFN-γ-responsive cell regulating neutrophil migration into the CNS. This question is best addressed in IFNGR^−∕−^ chimeras or mice with a cell-specific deficiency in the IFNGR. Because Gr-1 recognizes both Ly6C and Ly6G, only those papers identifying neutrophils using Ly6G are discussed. When IFNGR signaling was inhibited in a dominant negative manner leading to chronic EAE, no difference in neutrophil numbers was detected in either the acute or chronic phase of disease (Hindinger et al., 2012). Likewise, in the paper by Lee and colleagues in which the classical disease scores were similar in all four chimeras (WT → WT, IFNGR^−∕−^ → IFNGR^−∕−^, WT → IFNGR^−∕−^, IFNGR^−∕−^ → WT) there was no difference in the number of infiltrating neutrophils in the CNS. When we addressed this question in our chimera system in which IFNGR^−∕−^ → WT chimeras exhibit chronic progressive EAE (Figure 1A), we did not observe a difference in the total number of mononuclear cells (Figure 2A), CD4^+^ T cells (Figure 2B) or CD11b^+^ myeloid cells (Figure 2C) on day 10 (onset), 15 (peak) or 21 (recovery) after EAE induction in the two chimeras. However, when we observed the light scatter profiles an increase in high SSC cells consistent with granulocytes was evident in the IFNGR^−∕−^ → WT chimeras as compared to WT → WT chimeras (Figure 2D). We then utilized Ly6C and Ly6G to differentiate monocytes/macrophages (CD11b^+^Ly6C^+^Ly6G^−^) and neutrophils (CD11b^+^Ly6C^+^Ly6G^+^) (Figure 2E). When we quantitated cell numbers, we found that the IFNGR^−∕−^ → WT chimeras harbored significantly more neutrophils in the CNS on days 10 and 15 after EAE induction (Figure 2F). We then differentiated between the Ly6C^lo^ and Ly6C^hi^ monocyte/macrophage populations and found that only the Ly6C^lo^ population was significantly increased on day 10 (Figures 2G,H). The Ly6C^hi^ population consists of CCR2^+^ inflammatory macrophages (Geissmann et al., 2003). The Ly6C^lo^ population is comprised of microglial cells and non-inflammatory or resident macrophages. When we examined lesions in the chimera mice by immunofluorescence (Shriver and Dittel, 2006), as expected, we were able to identify lesions containing large numbers of CD11b^+^ myeloid cells in the spinal cord (blue) that also contained T cells (TCRβ, green) and neutrophils (red) in both chimeras (Figure 3). While we observed lesions in the brain stem in both chimeras, only the IFNGR^−∕−^ → WT chimeras contained large numbers of neutrophils (Figure 3). Given that only the peripheral immune system is devoid of IFNGR signaling in the IFNGR^−∕−^ → WT chimeras, these data indicate that peripheral immune cells are a major regulator of the influx of neutrophils into the brain stem. Whether this is due to production of ELR^+^ CXC chemokines by infiltrating cells is not known, but is plausible given that macrophages have been reported to produce CXCL1 and CXCL2 (Kanayama et al., 2015). Other studies have identified astrocytes and NG2^+^ glial cells as sources of ELR^+^ CXC chemokines, particularly in response to IL-17 signaling (Kang et al., 2010, 2013; Simmons et al., 2014; Ding et al., 2015).

## A role for neutrophils in MS?

While mouse studies in EAE clearly demonstrate an essential role for granulocytes (neutrophils) in EAE pathogenesis this has been more difficult to demonstrate in MS. Established MS lesions are largely devoid of neutrophils prompting many to conclude that neutrophils do not play a role in MS. However, the paucity of neutrophils in MS lesions is likely due the fact that neutrophils are short-lived once they enter tissues and undergo oxidative burst and degranulate. Little is known regarding neutrophil function in MS patients. While one study reported that neutrophils from MS patients exhibited depressed functions (Podikoglou et al., 1994), this finding was not consistent with two other reports demonstrating that neutrophils expressing a primed phenotype were increased in the peripheral blood of MS patients (Ziaber et al., 1998; Naegele et al., 2012). Recently, it was shown that the neutrophil-to-lymphocyte ratio was significantly higher in MS patients as compared to controls, with the ratio being the highest in MS patients in relapse (Demirci et al., 2015). In addition, logistic regression analysis with dichotomous expanded disability status scale (EDSS) score, which measures MS disability, revealed that a high neutrophil-to-lymphocyte ratio was a predictor of disability progression (Demirci et al., 2015). Additional evidence supporting a role for neutrophils in MS patients is their increase in serum CXCL8 (IL-8), which is a chemoattractant for both monocytes and neutrophils in humans (Lund et al., 2004). In a second report, elevated plasma levels of CXCL5, an ELR^+^ CXC chemokine, was reported in relapsing MS during new lesion formation (Rumble et al., 2015). Of importance, is that new lesion formation was not associated with increased levels of the lymphocyte and monocyte chemoattracting CXCL10 or CCL2, respectively (Rumble et al., 2015). In this same study, plasma levels of CXCL1, CXCL5 and neutrophil elastase directly correlated with the EDSS score and the cumulative MRI lesion volume on T1 weighted sequences indicating tissue damage and axonal loss (Rumble et al., 2015). In addition, CXCL5 and neutrophil elastase expression correlated with T2 weighted sequences that measure overall brain volume (Rumble et al., 2015). These findings in MS patients are consistent with the finding by the same group that neutrophils are essential to relapse in SJL/J mice (Carlson et al., 2008). More recently postmortem tissue from a MS patient affected by relapse following cessation of natalizumab therapy showed a prominent neutrophil presence in areas of BBB leakage (Aube et al., 2014). Overall, the above cumulative studies provide strong evidence that neutrophils play a pathogenic role in MS relapse. However, neutrophils are not necessarily a prerequisite for MS as demonstrated by two case reports in which MS patients exhibited low neutrophil counts due to either autoimmune neutropenia or a long-standing idiopathic neutropenia (Kozuka et al., 2003; Munzel et al., 2013). While the mechanism whereby neutrophils contribute to MS pathogenesis are not clearly understood, they likely involve their generation of reactive oxygen species that induce oxidative stress, which is considered a major driver of MS pathogenesis (Gilgun-Sherki et al., 2004).

## Concluding remarks

Since the failure of IFN-γ as a therapeutic treatment for MS, we have uncovered much about the biology of IFN-γ and its receptor. It is now appreciated that within the confines of the CNS, the anti-inflammatory properties of IFN-γ largely outweigh its pro-inflammatory mechanisms. Given the pleiotropic effects of IFN-γ and the fact that its receptor is expressed by virtually all cell types, in retrospect, it is not surprising that its systemic administration failed to have therapeutic benefit in MS. In the many EAE studies conducted in IFN-γ^−∕−^ and IFNGR^−∕−^ mice using a multitude of animal models it has become clear that one major mechanism whereby IFN-γ controls the severity and nature of EAE is by regulating the recruitment of neutrophils into the CNS. Specifically, we propose that encephalitogenic T cells enter the CNS and via their production of IL-17 and other inflammatory mediators induce the expression of ELR^+^ CXC chemokines by infiltrating macrophages and CNS resident astrocytes and NG2^+^ glial cells. Neutrophils in turn migrate into the CNS and produce oxygen radicals leading to the opening of the BBB and tissue damage. IFN-γ production by polarized Th1 cells or Th17 cells that have converted to Th1 cells directly downregulate the expression of ELR^+^ CXC chemokines restricting neutrophil migration into the CNS. These data also provide strong evidence that neutrophils must play an essential role in the pathogenesis of MS. Given that neutrophils can modulate disease by a variety of mechanisms including their well-established role in disruption of the BBB they should be considered as a therapeutic target in MS. In particular, the targeting of neutrophils during relapses is well justified.

### Conflict of interest statement

The authors declare that the research was conducted in the absence of any commercial or financial relationships that could be construed as a potential conflict of interest.
